# Association of PSA variability with prostate cancer development using large-scale medical information data: a retrospective cohort study

**DOI:** 10.1186/s41021-023-00280-7

**Published:** 2023-10-17

**Authors:** Ayako Maeda-Minami, Tomoki Nishikawa, Hideki Ishikawa, Michihiro Mutoh, Kazunori Akimoto, Yutaka Matsuyama, Yasunari Mano, Hiroji Uemura

**Affiliations:** 1 Faculty of Pharmaceutical Sciences, Tokyo University of Science, Yamazaki, Noda, 2641, 278-8510 Chiba Japan; 2 Department of Molecular-Targeting Prevention, Graduate School of Medical Science, Kyoto Prefectural University of Medicine, Kyoto, Japan; 3 Department of Biostatistics, School of Public Health, The University of Tokyo, Tokyo, Japan; 4 Yokohama City University Medical Center, Yokohama, Japan

**Keywords:** Prostate cancer, Prostate specific Antigen, Claim database, Retrospective cohort study, Real-word data

## Abstract

**Background:**

Prostate cancer is one of the most common cancers among men worldwide and the fourth most common cause of death. The number of prostate cancer cases and deaths is increasing every year because of population aging. This study aimed to clarify the risk of developing prostate cancer due to fluctuations in Prostate Specific Antigen (PSA) levels in patients without a history of prostate cancer using large medical information data.

**Results:**

This retrospective cohort included 1707 male patients aged 60 years or older who had a PSA level measurement date (2-PSA) within 3 months or more and 2 years from the first PSA level measurement date (1-PSA) in the database between 2008 and 2019. We subtracted 1-PSA from 2-PSA and designated patients with a higher 2-PSA than 1-PSA to the “up” group (n = 967) and patients with a lower 2-PSA than 1-PSA to the “down” group (n = 740). By using Cox proportional hazards model, a significant increase in prostate cancer risk was observed in the up group compared with the down group (adjusted hazard ratio [HR] = 1.82, 95% confidence interval [CI] = 1.21–2.72; adjusted for patient background factors). Subgroup analysis showed that patients with PSA levels < 4 ng/mL had a significantly increased risk of developing prostate cancer if the next PSA level increases by approximately 20% (adjusted HR = 2.94, 95% CI = 1.14–7.58), and patients with PSA levels of 4 ng/mL or higher if the next PSA level is decreased by approximately 20% had a significantly reduced risk of developing prostate cancer (adjusted HR = 0.36, 95% CI = 0.18–0.74), compared to that with no change.

**Conclusions:**

This is the first study to clarify the association between PSA variability and risk of developing prostate cancer in patients without a history of prostate cancer. These results suggest that the suppression of elevated PSA levels may lead to the prevention of prostate cancer and that it would be better to perform a biopsy because the risk of developing prostate cancer may increase in the future if the PSA value increases above a certain level.

**Supplementary Information:**

The online version contains supplementary material available at 10.1186/s41021-023-00280-7.

## Introduction

In 2020, prostate cancer was the second most common cancer among men worldwide and the fourth most common cause of death [1, 2]. The number of prostate cancer cases and deaths is increasing every year because of population aging [2–4]. In Japan, the number of patients with prostate cancer was the highest among male cancers in 2021 [5], and the age-adjusted incidence rate is increasing year by year [6]. The prevention of prostate cancer is an urgent issue in the future.

Prostate Specific Antigen (PSA) is a tumor marker for prostate cancer. PSA is an enzyme produced by the prostate gland and is detected in the blood under normal conditions; however, its concentration in the blood is known to be increased in prostate cancer and benign prostatic hyperplasia (BPH) cases [7, 8]. Prostate cancer screening using PSA is the first step for early detection, and the cut-off value for PSA testing has been set at 4.0 ng/mL in Japan [9]. Patients with PSA levels above the cut-off are advised prostate biopsy for a definitive diagnosis [9]. The higher the PSA level, the higher the rate of prostate cancer detection on biopsy [10].

An elevated PSA level after radical total prostatectomy or radical radiotherapy for prostate cancer is considered a biochemical recurrence [9, 11–14]. However, few studies have examined whether subsequent increase in PSA levels among patients with PSA levels below 4.0 ng/mL increase the risk of developing prostate cancer. There is also insufficient evidence regarding the incidence of subsequent prostate cancer in patients with a PSA level ≥ 4.0 ng/mL on their first PSA test but do not have prostate cancer. The current study aimed to clarify the risk of developing prostate cancer due to fluctuations in PSA levels in patients without a history of prostate cancer using a large medical information data.

## Materials and methods

### Database and study population

We used a large Japanese claim database of 28.44 million people held by Medical Data Vision Corporation (MDV). The database included data on healthcare costs, prescription drugs (date, dosage, days prescribed), attributes (e.g., age and sex), disease names coded by the International Classification of Disease 10th Revision (ICD-10), and laboratory data (PSA values). Prescribed drugs were coded according to the World Health Organization Anatomical Therapeutic and Chemical (ATC) classification.

**Fig. 1:**
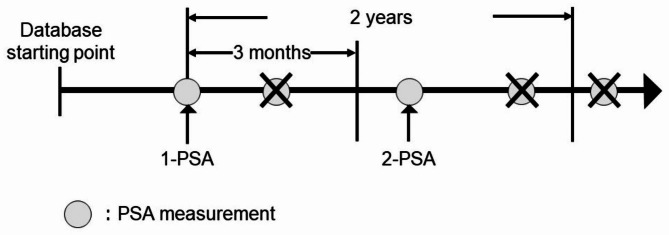
Study design Note: 1-PSA, PSA value that exists for the first time in the database; 2-PSA, PSA value measured for the first time within three months or more and two years after 1-PSA The PSA measurements “×” were not adopted because they met the exclusion criteria

A total of 2,066,100 men with at least 2 years of medical data from April 2008 to June 2019 and aged 60 years or older were included in this study. The exclusion criteria were patients who do not have a PSA level measurement date (2-PSA) within three months or more and two years from the first PSA level measurement date (1-PSA) in the database (Fig. 1), have no medical records for one year before the 1-PSA, were prescribed an antiandrogen drug (ATC group: gestanolone caproate G03DA01, chlormadinone G03DB06, allylestrenol G03DC01) before the 2-PSA, were diagnosed with prostate cancer or other cancers (ICD − 10 codes: C00-97) before the 2-PSA, and were diagnosed with prostatic-related disease (ICD-10 codes: N40-42,N510) before 2-PSA [15].

This research was conducted by following the Declaration of Helsinki, and the study protocol was approved by the appropriate institutional review board of Tokyo University of Science (approval no. 19,029).

### Patient characteristics

For the patient background of the covariates, age at 1-PSA and medical history (heart disease, peripheral vascular disease, cerebrovascular disease, hepatic disease, diabetes, renal disease, depression, and dyslipidemia) within one year before 1-PSA were obtained (Supplementary Table S1) [16–19].

### Relationship between PSA values up-down and prostate cancer risk

To confirm PSA variability in each patient, we subtracted 1-PSA from 2-PSA and designated patients with a higher 2-PSA than 1-PSA as an up group and patients with a lower 2-PSA than 1-PSA as a down group. Next, we compared the risk of developing prostate cancer in the up group and down group. Subsequently, subgroup analyses were performed for the patients with 1-PSA and 2-PSA levels greater than or less than 4.0 ng/mL and the patients in the gray zone with 1-PSA between 4 and 10 [9].

### Relationship between PSA value fluctuation rate and prostate cancer risk using cut-off values

The PSA value fluctuation rate (percentage change from 1-PSA to 2-PSA) was calculated as (2-PSA − 1PSA)/1-PSA. To calculate the cut-off value for the presence or absence of prostate cancer, the receiver operating characteristic (ROC) curves were generated using the PSA value fluctuation rate to calculate the cut-off value for patients with a PSA value fluctuation rate less than zero and greater than or equal to zero, respectively. The cut-off value that maximized the value of “Sensitivity − (1 - Specificity)” was calculated. Patients with a PSA value fluctuation rate above the cut-off value, calculated using only patients with a fluctuation rate of zero or more, were included in the increase group. Similarly, patients with a PSA value fluctuation rate below the cut-off value, calculated using only patients with a fluctuation rate of less than 0, were included in the decrease group. Patients who did not meet these conditions were selected as the reference group. The risk of prostate cancer in the increase and decrease groups was compared with that in the reference group. The subgroup analysis was then performed on the basis of 1-PSA values, with 1-PSA < 4.0 ng/mL, 1-PSA ≥ 4.0 ng/mL, and 4.0 ≤ 1-PSA < 10.0 ng/mL.

### Study outcomes and follow-up

The primary outcome was a diagnosis of prostate cancer (ICD-10 code: C61). Follow-up was started the day after the 2-PSA. The end of follow-up was defined as the earliest of (1) event occurrence, (2) end of the study period (June 2019), and (3) censoring for loss to follow-up. Patients with follow-up of less than one month were excluded.

### Statistical analysis

For the relationship between PSA values in the up and down groups and prostate cancer risk, Wilcoxon rank-sum tests were used to compare age, 1-PSA to 2-PSA period, 1-PSA, 2-PSA, and PSA value fluctuation rates of the up and down groups. The chi-square test was used to compare the medical history of the up and down groups. Up/down PSA values and the risk of developing prostate cancer were compared using Cox proportional hazards models to estimate crude and adjusted hazard ratios (HRs) and 95% confidence intervals (CIs). The adjustment factor was hepatic disease, which was significant in the comparison of age and background factors.

For the relationship between PSA value fluctuation rate and prostate cancer risk using cut-off values, the comparison of age between the reference group, increase group, and decrease group was performed by the steel test. The chi-square test was used for the comparison of medical history. PSA value fluctuation rate and prostate cancer risk were compared using Cox proportional hazards models to estimate crude and adjusted HRs and 95% CIs. The adjustment factors were age and depression, which were significant in the comparison of background factors. PSA value fluctuation rate and prostate cancer risk were compared using Cox proportional hazards models to estimate crude and adjusted HRs and 95% CIs. The adjustment factors were age and depression, which were significant in the comparison of background factors. A p-value < 0.05 was considered statistically significant. Statistical analysis was performed using SAS 9.4 (SAS institute, Cary, NC) and R software version 4.1.0 (The R Foundation for Statistical Computing, Vienna, Austria).

## Results

### Patient characteristics

**Fig. 2:**
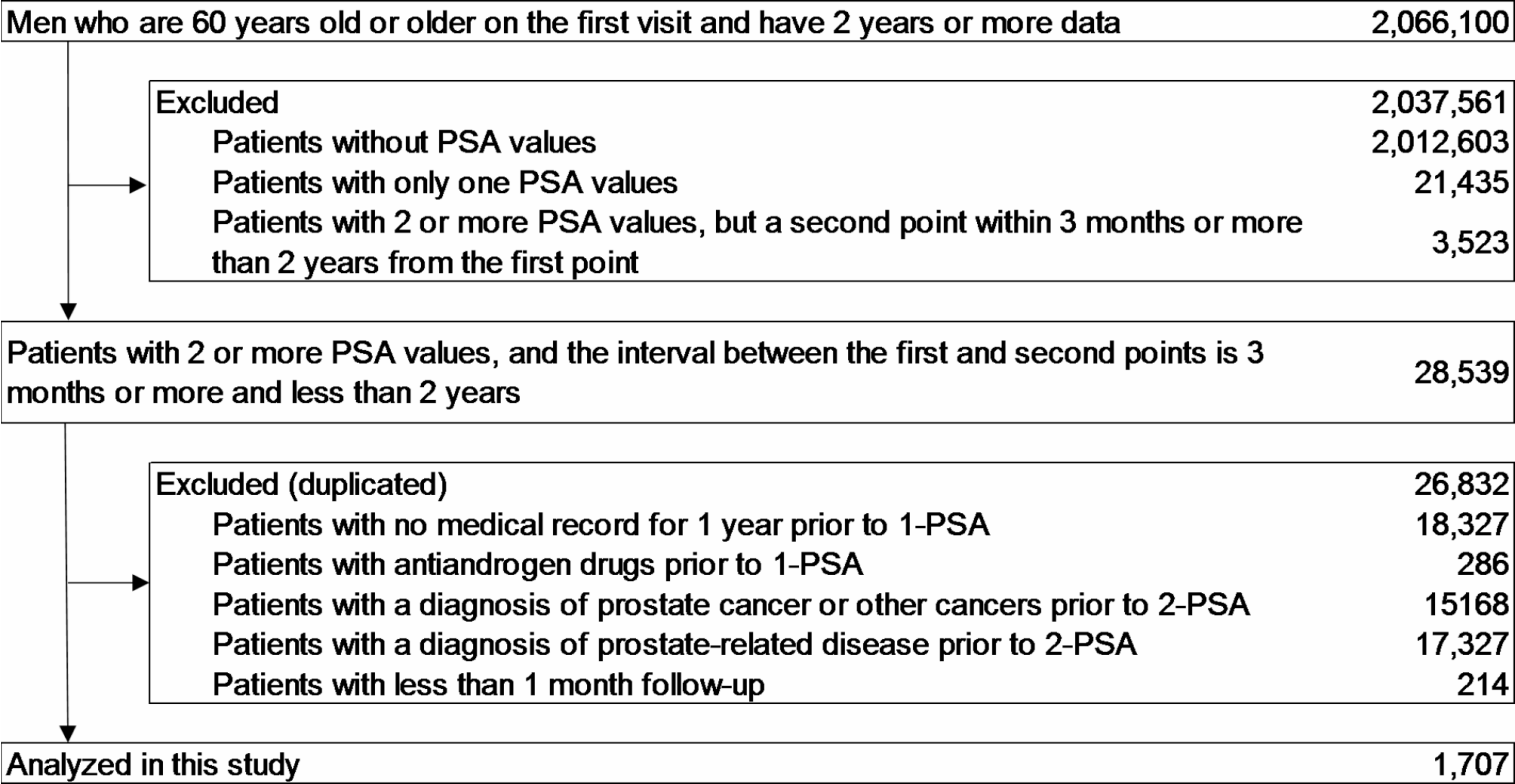
Exclusion flow Note: 1-PSA, PSA value that exists for the first time in the database; 2-PSA, PSA value measured for the first time within three months or more and two years after 1-PSA

The number of patients who had data on 2-PSA levels between 3 months and 2 years from 1-PSA during the study period was 28,539 patients in the MDV database (Fig. 2). After applying exclusion criteria, 1,707 patients were identified. The patient background is shown in Table 1. The median age was 73 years. Regarding medical history, hypertension was the most common at 57.2%, followed by dyslipidemia at 40%.

**Table 1:** Patient characteristics

Number of patients	1,707
Age (years)	73 (61–95)
Cardiovascular disease	567 (33.2)
Peripheral vascular disease	312 (18.3)
Cerebrovascular disease	356 (20.8)
Hepatic disease	225 (13.2)
Diabetes mellitus	627 (36.7)
Renal disease	239 (14.0)
Depression	49 (2.9)
Hypertension	976 (57.2)
Hyperlipidemia	682 (40.0)

Values are expressed as numbers (%) or medians (range)

### Relationship between PSA values up-down and prostate cancer risk

After subtracting 1-PSA from 2-PSA, and identifying the up and down groups, there were 967 patients in the up group and 740 patients in the down group. After comparing the up and down groups in terms of patient background, we found that the percentage of hepatic disease was significantly higher in the down group than in the up group (Supplementary Table S2). The evaluation period from 1-PSA to 2-PSA was significantly longer in the up group than in the down group. The 2-PSA was significantly higher in the up group than in the down group, and the 1-PSA was significantly higher in the down group than in the up group (Supplementary Table S3). The association between PSA values in up-down and the risk of developing prostate cancer is shown in Table 2. The number of patients who developed prostate cancer was 79 in the up group and 33 in the down group. The incidence of prostate cancer was 33.4 per 1000 person-years in the up group and 19.4 per 1000 person-years in the down group. After adjusting for covariates, the results of the Cox proportional hazards model showed a significantly higher risk of developing prostate cancer in the up group compared to the down group (adjusted HR = 1.82, 95% CI = 1.21–2.72).

**Table 2:** Relationship between PSA value changes and prostate cancer risk

Group	Total follow-up duration (person-years)	No. of prostate cancer cases	Incidence rate of prostate cancer (case/1,000 person-years)	Unadjusted HR (95% CI)		Adjusted HR (95% CI)	
Up (n = 967)	2,369	79	33.3	1.79 (1.20–2.69)	**	1.82 (1.21–2.72)	**
Down (n = 740)	1,704	33	19.4	1		1	

Adjusted for age, past medical history (hepatic disease)

**p < 0.01

Note: HR: hazard ratio; CI: confidence interval

The subgroup analysis of PSA levels divided into groups of ≥ 4.0 ng/mL and < 4.0 ng/mL showed that patients with 1-PSA ≥ 4.0 ng/mL (groups a, d, and e) and those with 1-PSA < 4.0 ng/mL but with 2-PSA ≥ 4.0 ng/mL (group b) had a significantly higher incidence of prostate cancer than those with 1-PSA and 2-PSA of < 4.0 ng/mL (group f) (Supplementary Table S4). As a result of subgroup analysis by 1-PSA value, in patients with 4.0 ng/mL ≤ 1-PSA < 10.0 ng/mL, the incidence of prostate cancer was significantly higher in patients with elevated 2-PSA (groups i and ii) than in those with PSA falling below 4.0 ng/mL (group iv) (Supplementary Table S5).

### Relationship between PSA value fluctuation rate and prostate cancer risk using cut-off values

After ROC analysis to calculate the cut-off value of PSA fluctuation rate on the basis of the presence or absence of prostate cancer, the cut-off values was − 0.176 for patients with a PSA value fluctuation rate < 0 and was 0.180 only for patients with a PSA value fluctuation rate ≥ 0. Patients were divided into three groups on the basis of these cut-off values of the PSA value fluctuation rate: The increase group consisted of patients who had a PSA values fluctuation rate ≥ 0.180 (patients with 2-PSA ≥ 18.0% higher than 1-PSA), the decrease group consisted of patients who had a PSA values fluctuation rate ≤ -0.176 (patients with ≥ 17.6% decline in 2-PSA compared with 1-PSA), and the reference group consisted of patients with PSA value fluctuation rate < 0.180 and greater than − 0.176 (patients with no or little change between 1-PSA and 2-PSA). The number of patients was 475, 318, and 914 among the increase, decrease, and reference groups, respectively. There was a significant difference in the proportion of patients with depression in the three groups (Supplementary Table 6). The PSA value fluctuation rate and the risk of developing prostate cancer are shown in Table 3. The number of patients who developed prostate cancer was 41 in the increase group, 60 in the reference group, and 11 in the decrease group. The incidence of prostate cancer was 36.1 per 1000 person-years in the increase group, 27.2 per 1000 person-years in the reference group, and 15.0 per 1000 person-years in the decrease group. After adjustment for covariates, Cox proportional hazards models showed no significant difference in the risk of prostate cancer in the increase group compared with the reference group (adjusted HR = 1.34, 95% CI = 0.90–1.99). After adjusting for covariates, the Cox proportional hazards models showed no significant difference in the risk of prostate cancer in the decrease group compared with the reference group (adjusted HR = 0.56, 95% CI = 0.30–1.07).

**Table 3:** Relationship between PSA value fluctuation rate and prostate cancer risk

1-PSA	Group	Total of follow-up period (person-years)	No. of onset of prostate cancer (case)	Incidence rate of prostate cancer (case/1,000 person-years)	Unadjusted HR (95% CI)		Adjusted HR (95% CI)	
All	Increase (n = 475)	1135	41	36.1	1.32 (0.89–1.97)		1.34 (0.90–1.99)	
	Reference (n = 914)	2205	60	27.2	1		1	
	Decrease (n = 318)	733	11	15.0	0.55 (0.29–1.04)		0.562 (0.30–1.07)	
4 ng/mL > 1-PSA	Increase (n = 361)	955	11	11.5	2.91 (1.13–7.51)	*	2.94 (1.14–7.58)	*
	Reference (n = 656)	1781	7	3.9	1		1	
	Decrease (n = 214)	517	2	3.9	0.98 (0.20–4.72)		0.94 (0.20–4.53)	
4 ng/mL ≤ 1-PSA	Increase (n = 114)	180	30	166.6	1.36 (0.87–2.14)		1.43 (0.91–2.26)	
	Reference (n = 258)	424	53	125.0	1		1	
	Decrease (n = 104)	216	9	41.7	0.35 (0.17–0.71)	**	0.36 (0.18–0.74)	**
4 ng/mL ≤ 1-PSA < 10 ng/mL	Increase (n = 97)	166	20	120.4	1.18 (0.69–2.03)		1.31 (0.75–2.26)	
	Reference (n = 222)	373	38	101.9	1		1	
	Decrease (n = 70)	161	4	24.9	0.26 (0.09–0.73)	*	0.27 (0.10–0.76)	*

Adjusted for age, past medical history (hepatic disease)

**p < 0.01, *p < 0.05

Note: HR: hazard ratio; CI: confidence interval; 1-PSA: PSA value that exists for the first time in the database

Subgroup analysis showed that patients with a 1-PSA of less than 4.0 ng/mL had a significantly increased risk of developing prostate cancer in the increase group compared with the reference group (adjusted HR = 2.94, 95% CI = 1.14–7.58). Patients with a 1-PSA ≥ 4.0 ng/mL had a significantly lower risk of developing prostate cancer in the decrease group than in the reference group (adjusted HR = 0.36, 95% CI = 0.18–0.74). Patients with a 1-PSA between 4.0 and 10.0 ng/mL had a significantly lower risk of developing prostate cancer in the decrease group than in the reference group (adjusted HR = 0.27, 95% CI = 0.10–0.76).

## Discussion

In this study, the analysis of a large medical information data suggested that in patients without a history of prostate cancer and patients with PSA levels higher than the first measured PSA level had a significantly increased risk of developing prostate cancer compared with those with PSA levels lower than the first measured PSA level. Furthermore, among patients with a 1-PSA < 4.0 ng/mL, those with an increase of 18% or more from 1-PSA to 2-PSA had a significantly higher risk of developing prostate cancer than those with no changes between 1-PSA and 2-PSA. Among patients with a 1-PSA ≥ 4 ng/mL, those with a decrease of approximately 18% or more from 1-PSA to 2-PSA had a significantly lower risk of developing prostate than those with no change between 1-PSA and 2-PSA. To date, PSA values have been used to determine prostate biopsy performance by using factors such as PSA > 4 ng/mL, PSA free/total ratio, PSA density, and PSA doubling time [20–23]. In the current study, when the level of PSA value was set to 4 ng/mL and after clarifying the subsequent increase and decrease in PSA dynamics, a significant difference in the incidence of prostate cancer was observed. We believe that the findings obtained in our study will enable non-specialists to more easily make decisions regarding referrals to a specialist.

Elevation in blood PSA levels might be a two-step process. The first is the promotion of PSA transcription. The androgen receptor (AR) of the prostate gland is translocated to the nucleus when dihydroxy testosterone binds. Thereafter, the ARs bind to androgen-responsive elements in the promoter regions of target genes, thus promoting the transcription of PSAs [24]. The second is the leakage of PSA into the blood vessels. The loss of glandular structure in the prostate gland due to prostate cancer or BPH allows PSA to enter the surrounding blood vessels [7, 8]. The process of prostate cancer development and malignant transformation is due to the overexpression of androgen-responsive genes in the PSA transcription-promoting cascade [25–29]. These support the findings of this study because elevated PSA levels are associated with an increased risk of prostate cancer.

For patients with 1-PSA < 4.0 ng/mL, an increase in 2-PSA of 18% or more over 1-PSA was associated with a significantly increased risk of developing prostate cancer compared with the group with no PSA variation (Table 3). The incidence of prostate cancer when 1-PSA is less than 4.0 ng/mL is 6.1 per 1000 person-years (calculated using the incidence rate of prostate cancer of group c and f in Supplementary Table S4 [data not shown]), and the incidence of prostate cancer when 2-PSA is higher than 1-PSA is 66.9 per 1000 person-years, which is approximately 10 times higher (Supplementary Table S4). Even if the 1-PSA is less than 4.0 ng/mL, a biopsy should be considered in case of an increase of 18% or more, as the risk of developing prostate cancer increases approximately threefold (Table 3).

For patients with 1-PSA > 4.0 ng/mL, a decrease in 2-PSA of approximately 18% or more was associated with a significantly lower risk of developing prostate cancer. Although a lower PSA level after radical treatment is associated with a better prognosis in patients with prostate cancer [9, 14], the current study is the first study to show that decreasing 2-PSA compared with 1-PSA in patients without a history of prostate cancer decreases the risk of prostate cancer. The detection rate of prostate cancer in Japanese subjects with PSA levels ≤ 2.0, 2.1–4.0, 4.0–10.0, and ≥ 10.0 ng/mL is 4.6%, 8.6%, 15.8%, and 59.5%, respectively; however, the specificity is not high [10]. In particular, there are concerns about the impaired quality of life due to excessive testing in gray zone patients with PSA levels of 4.0 to 10.0 ng/mL [9]. Therefore, in patients with 1-PSA levels in the gray zone, biopsies may be better performed in those patients whose 2-PSA did not decrease.

This research had several limitations. First, the PSA levels in this study were measured at the hospital; therefore, it is possible that many patients may have been tested because their doctors suspected they had prostate cancer. The results of this study may not be directly applicable to PSA values obtained from health examinations. The incidence of prostate cancer in Japan is reported to be 3.2 cases per 1000 person-years in men aged 65–69 years and 5.2 cases per 1000 person-years in men aged 70–74 years [5]. This is generally consistent with the incidence of prostate cancer in the patient group with an initial PSA < 4.0 ng/mL in the current study. Therefore, the analysis using this database might be valid. Second, we excluded patients who were prescribed antiandrogens, which are known to affect PSA levels. Other drugs that may affect PSA are unknown. Third, patients with a history of BPH and cancers other than prostate cancer were excluded from this study. Although BPH increases PSA levels, there is controversy over whether it is a risk factor for prostate cancer [29, 30]. A similar adjusted HR was obtained when other cancer types and prostatic hyperplasia were not excluded (data not shown). Fourth, in the current study, we considered the patient’s age and medical history as covariates, but other factors that affect PSA value, prostate cancer, and BPH cannot be considered covariates because we could not obtain them from big data. Genetic and environmental factors are known to influence PSA value, prostate cancer, and BPH. Regarding genetic factors, we reported that the prevalence of mutations in homologous recombination repair pathway genes in Japanese patients with metastatic castration-resistant prostate cancer was 35.7% [31]. Germline mutations and somatic mutations in *BRCA1* and *BRCA2* gene mutations account for approximately 6–7% each in prostate cancer; however, they do not strongly affect PSA values [31]. Although studies have been conducted on the genomic analysis of BPH, there is still no unified view [32, 33]. Regarding environmental factors, studies indicated that diet, obesity, smoking, and exercise are associated with the development of prostate cancer [34–36], and similar reports have been published for BPH [37, 38]. Although PSA values increase with age, there is no consensus regarding the influence of other environmental factors [39]. Another factor that affects prostate cancer development is the comorbidity of BPH [40–42]. Further studies are needed to evaluate the effect of BPH on prostate cancer.

The association between changes in PSA levels and the development of prostate cancer in patients without a history of prostate cancer showed that the probability of developing prostate cancer increased in patients with PSA levels higher than first measured using a large medical information database. In addition, even in patients with PSA level < 4.0 ng/mL, if the next PSA level increases by approximately 20%, our findings suggested that it would be better to perform a biopsy because the risk of developing prostate cancer may increase in the future. Furthermore, even in patients with PSA level ≥ 4.0 ng/mL, it was suggested that the risk of developing prostate cancer may be reduced if the following PSA levels are decreased by approximately 20%. These results suggest that the suppression of elevated PSA levels may lead to the prevention of prostate cancer. Therefore, the findings of this study may help clinicians better predict the risk of developing prostate cancer and employ preventive strategies.

### Electronic supplementary material

Below is the link to the electronic supplementary material.

Supplementary Material 1

## Data Availability

Data that support the findings of this study are available from Medical Data Vision Corporation, but restrictions apply to the availability of these data, which were used under license for the current study. The data are available from the authors upon reasonable request and with the permission of Medical Data Vision Corporation.

## References

[1] Cancer Today. World Health Organization. 2020. https://gco.iarc.fr/today/home. Accessed 16 September 2021.

[2] Sung H, Ferlay J, Siegel RL, Laversanne M, Soerjomataram I, Jemal A (2021). Global cancer statistics 2020: GLOBOCAN estimates of incidence and mortality worldwide for 36 cancers in 185 countries. CA Cancer J Clin.

[3] Torre LA, Bray F, Siegel RL, Ferlay J, Lortet-Tieulent J, Jemal A (2015). Global cancer statistics, 2012. CA Cancer J Clin.

[4] Jemal A, Bray F, Center MM, Ferlay J, Ward E, Forman D (2011). Global cancer statistics. CA Cancer J Clin.

[5] Cancer Statistics Predictions. National Cancer Center Japan. 2021. http://ganjoho.jp/reg_stat/statistics/stat/short_pred.html. Accessed 16 September 2021.

[6] Katanoda K, Hori M, Saito E, Shibata A, Ito Y, Minami T (2021). Updated trends in cancer in Japan: incidence in 1985–2015 and mortality in 1958–2018-A sign of decrease in cancer incidence. J Epidemiol.

[7] Stenman UH, Leinonen J, Zhang WM, Finne P (1999). Prostate-specific antigen. Semin Cancer Biol.

[8] Balk SP, Ko YJ, Bubley GJ (2003). Biology of prostate-specific antigen. J Clin Oncol.

[9] Committee for Establishment of the Guidelines on Screening for Prostate Cancer, Japanese Urological Association (2010). Updated japanese Urological Association Guidelines on prostate-specific antigen-based screening for prostate cancer in 2010. Int J Urol.

[10] Egawa S, Matsumoto K, Yoshida K, Iwamura M, Kuwao S, Koshiba K (1998). Results of transrectal ultrasound-guided biopsies and clinical significance of japanese prostate cancer. Jpn J Clin Oncol.

[11] Ito K, Raaijmakers R, Roobol M, Wildhagen M, Yamanaka H, Schröder FH (2005). Prostate carcinoma detection and increased prostate-specific antigen levels after 4 years in dutch and japanese males who had no evidence of disease at initial screening. Cancer.

[12] Sawada K, Kitagawa Y, Ito K, Takeda Y, Mizokami A, Namiki M (2014). Cumulative risk of developing prostate cancer in men with low ( ≦ 2.0 ng/mL) prostate-specific antigen levels: a population-based screening cohort study in Japan. Int J Urol.

[13] McGreevy K, Rodgers K, Lipsitz S, Bissada N, Hoel D (2006). Impact of race and baseline PSA on longitudinal PSA. Int J Cancer.

[14] Roach M, Hanks G, Thames H, Schellhammer P, Shipley WU, Sokol GH (2006). Defining biochemical failure following radiotherapy with or without hormonal therapy in men with clinically localized prostate cancer: recommendations of the RTOG-ASTRO Phoenix Consensus Conference. Int J Radiat Oncol Biol Phys.

[15] Fujimoto K, Hirao Y, Masumori N, Arai Y, Yamanaka H, Kato T (2006). Prostate-specific antigen changes as a result of chlormadinone acetate administration to patients with benign prostatic hyperplasia: a retrospective multi-institutional study. Int J Urol.

[16] Wright FL, Green J, Canoy D, Cairns BJ, Balkwill A, Beral V (2012). Vascular disease in women: comparison of diagnoses in hospital episode statistics and general practice records in England. BMC Med Res Methodol.

[17] Thygesen SK, Christiansen CF, Christensen S, Lash TL, Sørensen HT (2011). The predictive value of ICD-10 diagnostic coding used to assess Charlson comorbidity index conditions in the population-based danish National Registry of Patients. BMC Med Res Methodol.

[18] Gradus JL, Qin P, Lincoln AK, Miller M, Lawler E, Sorensen HT (2010). Posttraumatic stress disorder and completed suicide. Am J Epidemiol.

[19] Junqueira RMP, Duarte EC (2012). Hospitalizations due to ambulatory care-sensitive conditions in the Federal District, Brazil, 2008. Rev Saude Publica.

[20] Okihara K, Kitamura K, Okada K, Mikami K, Ukimura O, Miki T (2008). Ten year trend in prostate cancer screening with high prostate-specific antigen exposure rate in Japan. Int J Urol.

[21] Finne P, Auvinen A, Määttänen L, Tammela TL, Ruutu M, Juusela H (2008). Diagnostic value of free prostate-specific antigen among men with a prostate-specific antigen level of < 3.0 µg per liter. Eur Urol.

[22] Sasaki M, Ishidoya S, Ito A, Saito H, Yamada S, Mitsuzuka K (2014). Low percentage of free prostate-specific antigen (PSA) is a strong predictor of later detection of prostate cancer among japanese men with serum levels of total PSA of 4.0 ng/mL or less. Urology.

[23] Fujizuka Y, Ito K, Oki R, Suzuki R, Sekine Y, Koike H (2017). Predictive value of different prostate-specific antigen-based markers in men with baseline total prostate-specific antigen < 2.0 ng/mL. Int J Urol.

[24] Gottlieb B, Lombroso R, Beitel LK, Trifiro MA (2005). Molecular pathology of the androgen receptor in male (in)fertility. Reprod Biomed Online.

[25] Shang Y, Myers M, Brown M (2002). Formation of the androgen receptor transcription complex. Mol Cell.

[26] Kang Z, Jänne OA, Palvimo JJ (2004). Coregulator recruitment and histone modifications in transcriptional regulation by the androgen receptor. Mol Endocrinol.

[27] Wang Q, Carroll JS, Brown M (2005). Spatial and temporal recruitment of androgen receptor and its coactivators involves chromosomal looping and polymerase tracking. Mol Cell.

[28] Obinata D, Takayama K, Urano T, Murata T, Kumagai J, Fujimura T (2012). Oct1 regulates cell growth of LNCaP cells and is a prognostic factor for prostate cancer. Int J Cancer.

[29] Dai X, Fang X, Ma Y, Xianyu J (2016). Benign prostatic hyperplasia and the risk of prostate cancer and bladder cancer: a meta-analysis of observational studies. Med (Baltim).

[30] Kang D, Chokkalingam AP, Gridley G, Nyren O, Johansson JE, Adami HO (2007). Benign prostatic hyperplasia and subsequent risk of bladder cancer. Br J Cancer.

[31] Uemura H, Oya M, Kamoto T, Sugimoto M, Shinozaki K, Morita K (2023). The prevalence of gene mutations in homologous recombination repair pathways in japanese patients with metastatic castration-resistant prostate cancer in real-world clinical practice: the multi-institutional observational ZENSHIN study. Cancer Med.

[32] Hellwege JN, Stallings S, Torstenson ES, Carroll R, Borthwick KM, Brilliant MH (2019). Heritability and genome-wide association study of benign prostatic hyperplasia (BPH) in the eMERGE network. Sci Rep.

[33] Ke ZB, Cai H, Wu YP, Lin YZ, Li XD, Huang JB (2019). Identification of key genes and pathways in benign prostatic hyperplasia. J Cell Physiol.

[34] Matsushita M, Fujita K, Nonomura N (2020). Influence of Diet and Nutrition on prostate Cancer. Int J Mol Sci.

[35] Bostwick DG, Burke HB, Djakiew D, Euling S, Ho SM, Landolph J (2004). Timms B. Human prostate cancer risk factors. Cancer.

[36] Nomura AM, Kolonel LN (1991). Prostate cancer: a current perspective. Epidemiol Rev.

[37] Jung JH, Ahn SV, Song JM, Chang SJ, Kim KJ, Kwon SW (2016). Obesity as a risk factor for Prostatic Enlargement: a retrospective cohort study in Korea. Int Neurourol J.

[38] Araki H, Watanabe H, Mishina T, Nakao M (1983). High-risk group for benign prostatic hypertrophy. Prostate.

[39] Shibata A, Whittemore AS, Imai K, Kolonel LN, Wu AH, John EM (1997). Serum levels of prostate-specific antigen among japanese-american and native japanese men. J Natl Cancer Inst.

[40] Bergengren O, Pekala KR, Matsoukas K, Fainberg J, Mungovan SF, Bratt O (2023). 2022 update on prostate Cancer epidemiology and risk Factors-A systematic review. Eur Urol.

[41] Gandaglia G, Leni R, Bray F, Fleshner N, Freedland SJ, Kibel A (2021). Epidemiology and Prevention of prostate Cancer. Eur Urol Oncol.

[42] Miah S, Catto J (2014). BPH and prostate cancer risk. Indian J Urol.

