# Supplementary material for: Association of PSA variability with prostate cancer development using large-scale medical information data: a retrospective cohort study
Source: Genes Environ. 2023 Oct 17;45:25. doi: 10.1186/s41021-023-00280-7 (PMC10580524; doi:10.1186/s41021-023-00280-7)
Supplement: Supplementary file 1 — Supplementary Material 1 [file 41021_2023_280_MOESM1_ESM.docx]

Supplementary Table S1. ICD-10 codes of medical history.

| Disease name | ICD-10 code |
| --- | --- |
| Cardiovascular disease | I20–I25, I50, J81 |
| Peripheral vascular disease | I70–I74, I77 |
| Cerebrovascular disease | I60–I69, G45 |
| Hepatic disease | B150, B160, B162, B18, B190, K700–K704, K709, K71–K74, K760, K766, I85 |
| Diabetes mellitus | E10–E14 |
| Renal disease | I12, I13, N00–N05, N07, N11, N14, N17–N19, Q61 |
| Depression | F32, F33, F34.1 |
| Hypertension | I10, I11 |
| Hyperlipidemia | E78 |

Supplementary Table S2. Patient background of the up and down groups.

|  | Up group  (n = 967) | | Down group (n = 740) | |  |
| --- | --- | --- | --- | --- | --- |
| Age | 73 (62–95) | | 73 (61–95) | |  |
| Cardiovascular disease | 320 | (33.1) | 247 | (33.4) |  |
| Peripheral vascular disease | 179 | (18.4) | 133 | (18.0) |  |
| Cerebrovascular disease | 217 | (22.4) | 139 | (18.8) |  |
| Hepatic disease | 120 | (12.4) | 105 | (14.2) | * |
| Diabetes mellitus | 352 | (36.4) | 275 | (37.2) |  |
| Renal disease | 130 | (13.4) | 109 | (14.7) |  |
| Depression | 30 | (3.1) | 19 | (2.6) |  |
| Hypertension | 559 | (57.8) | 417 | (56.4) |  |
| Hyperlipidemia | 387 | (40.0) | 295 | (39.9) |  |

Values are expressed as numbers (%) or medians (range).

*p < 0.05

Supplementary Table S3. PSA values of the up and down groups.

|  | Up group (n = 967) | Down group (n = 740) |  |
| --- | --- | --- | --- |
| Period from 1-PSA to 2-PSA (day) | 336  (91–728) | 295  (91–728) | *** |
| 1-PSA (ng/mL) | 1.59  (0–50.7) | 1.99  (0–107) | *** |
| 2-PSA (ng/mL) | 1.97  (0–67.8) | 1.45  (0–79.8) | *** |
| PSA value fluctuation rate | 0.178 (0–30.3) | -0.148 (-0.998 to -0.001) |  |

Note: 1-PSA, PSA value that exists for the first time in the database; 2-PSA, PSA value measured for the first time within three months or more and two years after 1-PSA; PSA values fluctuation rate, (2-PSA - 1-PSA)/1-PSA.

***p < 0.001

Supplementary Table S4. Results of the subgroup analysis by PSA value.

| Group | | 1-PSA | 2-PSA | Number of patients | Total of  follow-up  period (person-years) | No. of  onset of  prostate cancer (case) | Incidence rate of prostate cancer (case/1,000  person-years) | Unadjusted HR (95% CI) |  | Adjusted HR (95% CI) |  |
| --- | --- | --- | --- | --- | --- | --- | --- | --- | --- | --- | --- |
| UP | a | ≤4≤ | | 248 | 400 | 62 | 155.0 | 56.3 (17.6–180) | *** | 58.8 (18.4–188) | *** |
|  | b | 4> | 4≤ | 43 | 90 | 6 | 66.9 | 28.0 (6.99–112) | *** | 67.4 (9.45–480) | *** |
|  | c | 4> | | 676 | 1879 | 11 | 5.9 | 2.46 (0.69–8.81) |  | 2.47 (0.69–8.87) |  |
| Down | d | 4≤ | | 104 | 185 | 18 | 97.3 | 38.9 (12.4–133) | *** | 37.6 (10.9–129) | *** |
|  | e | 4≤ | 4> | 124 | 236 | 12 | 50.8 | 21.5 (6.00–77.1) | *** | 23.1 (6.35–84.0) | *** |
|  | f | 4> | | 512 | 1284 | 3 | 2.3 | 1 |  | 1 |  |

Note: 1-PSA, PSA value that exists for the first time in the database; 2-PSA, PSA value measured for the first time within three months or more and two years after 1-PSA.

***p < 0.001

Supplementary Table S5. Results of the subgroup analysis by 1-PSA value.

| Group | | 1-PSA | 2-PSA | Number of patients | Total of  follow-up  period (person-years) | No. of  onset of  prostate cancer (case) | Incidence rate of prostate cancer (case/1,000  person-years) | Unadjusted HR (95% CI) |  | Adjusted HR (95% CI) |  |
| --- | --- | --- | --- | --- | --- | --- | --- | --- | --- | --- | --- |
| Up | i | ≤4  1-PSA  <10 | 10 ≤ 2-PSA | 32 | 51.5 | 9 | 174.8 | 5.19 (1.60–16.9) | ** | 4.50 (1.38–14.7) | * |
|  | ii |  | 4 ≤ 2-PSA  <10 | 180 | 308.6 | 33 | 106.9 | 2.72 (1.06–6.98) | * | 2.76 (1.06–7.17) | * |
| Down | iii |  | 4 ≤ 2-PSA  <10 | 118 | 190.3 | 15 | 78.8 | 2.31 (0.433–6.59) |  | 2.32 (0.79–6.78) |  |
|  | iv |  | 2-PSA ≤ 4 | 59 | 149.5 | 5 | 33.4 | 1 |  | 1 |  |

Note: 1-PSA, PSA value that exists for the first time in the database; 2-PSA, PSA value measured for the first time within three months or more and two years after 1-PSA.

**p < 0.01, *p < 0.05

Supplementary Table S6. Patient background of the up, reference, and down groups.

|  | Reference group (n = 914) | | Increase group (n = 475) | | Decrease group (n = 318) | |  |
| --- | --- | --- | --- | --- | --- | --- | --- |
| Age | 73 (61–95) | | 73 (62–95) | | 73 (61–95) | |  |
| Cardiovascular disease | 299 | (32.7) | 154 | (32.4) | 114 | (35.8) |  |
| Peripheral vascular disease | 161 | (17.6) | 93 | (19.6) | 58 | (18.2) |  |
| Cerebrovascular disease | 204 | (22.3) | 92 | (19.4) | 60 | (18.9) |  |
| Hepatic disease | 120 | (13.1) | 61 | (12.8) | 44 | (13.8) |  |
| Diabetes mellitus | 332 | (36.3) | 169 | (35.6) | 126 | (39.6) |  |
| Renal disease | 122 | (13.3) | 64 | (13.5) | 53 | (16.7) |  |
| Depression | 17 | (1.9) | 19 | (4.0) | 13 | (4.1) | * |
| Hypertension | 510 | (55.8) | 279 | (58.7) | 187 | (58.8) |  |
| Hyperlipidemia | 365 | (39.9) | 191 | (40.2) | 126 | (39.6) |  |

Values are expressed as numbers (%) or medians (range).

Age was tested using Steel’s multiple comparison test. Medical history was tested using Pearson’s chi-square test.

*p < 0.05
